# Impact of Ni Doping on the Microstructure and Mechanical Properties of TiB_2_ Films

**DOI:** 10.3390/nano15030229

**Published:** 2025-01-31

**Authors:** Ying Wang, Xu Wang, Hailong Shang, Xiaotong Liu, Yu Qi, Xiaoben Qi, Ning Zhong

**Affiliations:** 1 School of Materials Science, Shanghai Dianji University, Shanghai 201306, China; ywang@sdju.edu.cn (Y.W.); wx19980927@163.com (X.W.); shanghl@sdju.edu.cn (H.S.); dndxj@126.com (X.L.); m15135239996@163.com (Y.Q.); 2 College of Ocean Science and Engineering, Shanghai Maritime University, Shanghai 201306, China; ningzhong@shmtu.edu.cn

**Keywords:** TiB_2_ film, Ni doping, nanocomposite, microstructure, mechanical property

## Abstract

The TiB_2_ film exhibits exceptional hardness and chemical stability due to its unique crystal structure and robust covalent bonds, but it also demonstrates high brittleness and poor toughness, which restricts its practical applications in engineering. By appropriately incorporating metal dopants, the toughness of the ceramic matrix can be enhanced without compromising its inherent hardness. In this study, TiB_2_ films with different nickel contents (0–32.22 at.%) were fabricated through radio frequency magnetron sputtering. The microstructure, chemical composition, phase structure, and mechanical properties were analyzed using scanning electron microscopy, transmission electron microscopy, X-ray diffraction, X-ray photoelectron spectroscopy and nanoindentation tester. The pure TiB_2_ film exhibited (0001) and (0002) peaks; however, the addition of nickel resulted in broadening of the (0001) peak and disappearance of the (0002) peak, and no crystalline nickel or other nickel-containing phases could be identified. It was found that the incorporation of nickel refines the grain structure of titanium diboride, with nickel present in an amorphous form at the boundaries of titanium diboride, thereby forming a wrapped structure. The enrichment of nickel at the grain boundary becomes more pronounced as the nickel content is further increased, which hinders the growth of TiB_2_ grains, resulting in the thinning of columnar crystals and formation of nanocrystalline in the film, and the coating hardness remains above 20 GPa, when the nickel content is less than 10.83 at.%. With the increase in nickel content, titanium diboride exhibited a tendency to form an amorphous structure, while nickel became increasingly enriched at the boundaries, and the coating hardness and elastic modulus decreased. The wrapped microstructure could absorb the energy generated by compressive shear stress through plastic deformation, which should be beneficial to improve the toughness of the coatings. The addition of nickel enhanced the adhesion between the film and substrate while reducing the friction coefficient of the film. Specifically, when the nickel content reached 4.26 at.%, a notable enhancement in both nanohardness and toughness was observed for nanocomposite films.

## 1. Introduction

Due to the unique chemical bond composition of TiB_2_ ceramics, they exhibit exceptionally high hardness, melting point, and excellent chemical stability [1,2]. As a result, new TiB_2_-based coatings have been extensively developed. Various coating preparation technologies exist including laser cladding [3], thermal spraying [4], electrochemical deposition [5], chemical vapor deposition [6], physical vapor deposition [7], and more. Among these, sputtering technology is commonly used due to its low deposition temperature requirement and relatively high coating rates [8]. Researchers [9,10] have discovered that boron-rich TiB_x_ films with x typically ranging from 2.2 to 3.5 can be obtained using magnetron sputtering. Studies by Christian Mitterer [11] suggested that B atoms in the sputtered plasma have a smaller collision cross-sectional area and energy transfer coefficient compared to Ti atoms. This results in the B atoms being less likely to collide with Ar atoms during the plasma transport process leading to lower power loss after collisions and increased likelihood of B atoms being deposited on the substrate surface. In addition, high-power impact magnetron sputtering has been employed to produce highly understoichiometric TiB_x_ with x as low as ~1.4 [12]. Researchers [13] propose that excess Ti leads to planar stacking fault defects with islands where one or two boron planes are missing in the films. The disruption of crystalline structures inhibits dislocation propagation affecting mechanical properties. Moreover, it has been reported by numerous researchers that the hardness of the TiB_2_ coating is closely related to optimal orientation which can be regulated by parameters of magnetron sputtering. It is often observed that the high hardness of the TiB_2_ coating exhibits a strong (0001) preferred orientation [14,15].

However, the main drawback of TiB_2_ coatings is the low toughness, high residual stress, and low cracking resistance in the deposited films [16,17], making it difficult to achieve the desired mechanical properties. In addition to optimizing coating properties by adjusting deposition parameters, two other effective methods for improving the performance of hard films are through multilayer structures [18,19] and nanocomposites [20]. The structural coating with a periodic arrangement is achieved through alternating deposition, and its properties are influenced by both the multilayer period and the interface between adjacent layers. Douglas E. Wolfe [21] found that the stress of TiB_2_/TiC multilayers decreases with an increase in the number of layers, thereby enhancing the film toughness. O.N. Grigor’ev [22] prepared a TiB_2_ coating containing a β-SiC layer with a porous arch structure which eliminates crack formation due to its high relaxation capacity. The intricate influence of interface structure and interlayer in multilayer films can impede dislocation slip and crack propagation [23], thereby not only contributing to toughening but also enhancing other properties.

Element doping is an additional effective approach to modify the properties of the film, thereby exerting influence on its structure and phase composition, consequently impacting both hardness and toughness. The coexistence of the doped second phase is facilitated by the narrow phase interval of TiB_2_. The findings of these studies by adding Cu [24], Ni [25], Cr [26], C [27], W [28], Al [5], Zr [29] and high-entropy alloy elements [30] to form a nanocomposite structure demonstrate that the incorporation of dopant elements can effectively restrict the growth of TiB_2_ grains, leading to a reduction in grain size. The study conducted by Nedfors et al. [31] revealed that the incorporation of aluminum into the TiB_2_ coating resulted in an improved ratio of hardness to Young’s modulus, thereby enhancing its toughness, The presence of the second phase in nanocrystalline or amorphous form enables effective control over the plastic zone at the crack tip, impeding crack propagation and enhancing the coating′s toughness. The influence of adding Al, Ni, and Zr on the crystal structure and elastic constant of TiB_2_ coatings was theoretically analyzed using first principles by Xian Lijun [32], and the results indicated that when Ti atoms in the lattice of TiB_2_ are partially replaced by Al, Ni and Zr atoms, the cell volume and the binding energy of the (Ti_1−x_M_x_)B_2_ ternary coatings increases, which leads to a reduction in the hardness and the improvement of the coating toughness, and the addition of nickel has a more pronounced impact on the reduction in hardness of the TiB_2_ coating.

Due to the good wettability of nickel and TiB_2_, as well as the rapid diffusion rate of nickel within the hexagonal lattice [33,34], favorable conditions are created for the formation of TiB_2_-Ni nanocomposite films. The study of obtaining a film with both toughness and hardness is worth pursuing. In this study, TiB_2_ films with different nickel concentrations were deposited using radio frequency magnetron sputtering. The microstructure, chemical and phase composition, as well as the mechanical properties of Ni-doped TiB_2_ films, were characterized using X-ray diffraction, X-ray photoelectron spectroscopy, scanning electron microscopy with energy-dispersive X-ray spectroscopy, transmission electron microscopy, nanoindentation techniques and Tribology Test Equipment. Subsequently, the effects of different Ni content on the microstructure, nanohardness, toughness and wear performance of TiB_2_ coatings were systematically evaluated.

## 2. Materials and Methods

### 2.1. Film Deposition

TiB_2_ films with different nickel content were deposited onto single-crystal silicon substrates (10 mm × 10 mm × 1 mm) and using magnetron sputtering (ANAVA SPC-350, Anelva, Tokyo, Japan). A twin radio frequency current magnetron setup, equipped with disk-shaped targets made of pure nickel (Ni, ϕ 76 mm) and titanium diboride (TiB_2_, ϕ 76 mm), was employed for the deposition. After ultrasonic cleaning with acetone for 10 min, the substrate was subjected to constant temperature drying, followed by placement into the vacuum chamber and subsequent vacuuming to achieve a pressure of 3 × 10^−1^ Pa.

Radio frequency magnetron sputtering is particularly suitable for materials with poor electrical conductivity or insulating properties, such as ceramics and glass. Sputtering power, argon pressure, substrate rotation speed and substrate temperature are critical parameters that significantly influence the sputtering process. The sputtering power dictates the energy imparted to the target material, thereby influencing both the quantity and energy distribution of the sputtered particles. With the increase in power, the deposition rate increases, and when the power is excessively high, it can lead to the sputtered atoms arriving at the substrate surface with elevated energy levels. It induces significant internal stress within the film, thereby reducing the adhesion between the film and the substrate. Higher pressure typically results in greater plasma density, but it can also increase the frequency of collisions between sputtered particles, which may compromise the uniformity and quality of the deposited film. Conversely, lower air pressure facilitates the formation of high-energy sputtered particles, making it more suitable for applications that demand highly dense and uniform films. The substrate temperature plays a crucial role in determining the film structure, grain growth and binding strength. High temperature facilitates crystal growth and densification of the film, but it may also induce thermal deformation or damage to the substrate. It can be controlled directly through the substrate heating system or indirectly by adjusting the sputtering power and chamber pressure.

In this paper, the argon pressure in the vacuum chamber during sputtering was maintained at 0.5 Pa, while the power on the Ni target was adjusted within the range of 5 to 30 W in order to achieve different Ni contents. The power on the TiB_2_ target remained constant at 180 W throughout. The substrate rotated at a speed of 20 revolutions per minute during deposition, and each film was sputtered for a duration of 4 h. The composite sputtering deposition was achieved through the cyclic rotation of the substrate frame in front of the target, enabling the adjustment of target power to obtain TiB_2_-Ni films with varying amounts of Ni doping.

### 2.2. Film Characterization

Phase analyses of the films were conducted using X-ray diffraction with a Bruker D8 ADVANCE diffractometer (Brucker, Karlsruhe, Germany) equipped with a Cu K_ɑ_ lamp (0.154 nm). The cross-sectional morphology of the films after sputtering was observed using scanning electron microscopy (ZEISS Sigma300, ZEISS, Oberkochen, Germany) and transmission electron microscopy (JE-2010ARP, JEOL, Tokyo, Japan) by preparing cross-sectional lamella manufactured via focused ion beam (FIB). Selected area electron diffraction patterns, obtained using an ϕ 150 nm aperture, were used for phase analysis.

Additionally, the composition of the films was analyzed using an energy-dispersive spectrometer (Oxford Xplore30, Oxford Instruments, Oxford, UK). X-ray photoelectron spectroscopy (XPS) with a Thermo Fisher Scientific K-Alpha instrument (Thermo Fisher Scientific, Waltham, United States) was utilized for qualitative and quantitative analysis of the chemical bond state and phase structure of elements in the films. The Al K_ɑ_ source with the energy of 1486.6 eV was used, and the spot size was 400 μm.

The nanoindentation technique was employed to investigate the nanohardness of TiB_2_ films with different Ni contents, using Paar’s Step300-NTH3 instrument (Anton Paar, Graz, Austria) equipped with a Berkovich diamond tip. A maximum force of 10 mN was applied, with loading and unloading rates set at 30 mN/min. The values of hardness (H), elastic modulus (E), maximum indentation depth (h_max_), and indentation depth (h_p_) were analyzed through the load–displacement curve using the Oliver–Pharr method. Parameters such as H/E, H^3^/E^2^, and normalized plastic depth value (δ_H_) were calculated to evaluate the mechanical properties of the films, where the normalized plastic depth value is equivalent to the indentation depth (h_p_) divided by maximum indentation depth (h_max_). At least nine measurements were conducted for each load condition.

**Figure 1:**
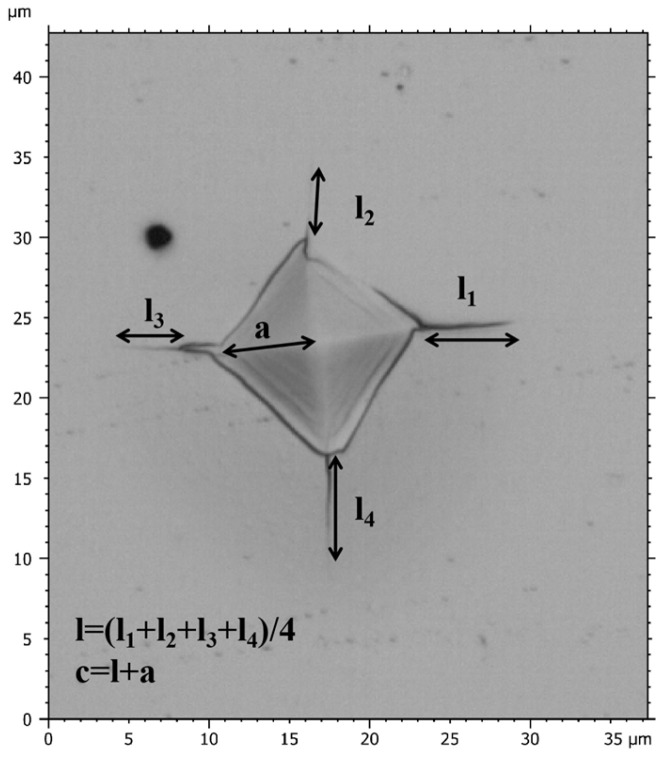
Schematic diagram of crack length (c) and half of the diagonal length of the indentation (a).

The fracture toughness of the films was determined using the Vickers indentation method, with a load of 500 mN applied to the film surface. After unloading, the fracture toughness can be calculated according to Equation (1) [35] by examining the indentation morphology and measuring the diagonal crack length of the indentation.

(1) $$K_{IC}=\delta {\left(\frac{a}{l}\right)}^{\frac{1}{2}}{\left(\frac{E}{H}\right)}^{\frac{2}{3}}(\frac{P}{c^{3/2}})$$

where E (GPa) and H (GPa) represent the elastic modulus and hardness of the films, respectively. P (mN) stands for Vickers indentation load. The empirical coefficient (δ) is 0.016 [36], and c (μm) denotes the total length from the center to the end of crack as shown in Figure 1, where a represents half of the diagonal length of the indentation, and l represents the average length of cracks. The indentation morphology of films was observed using a microscope (Leica DCM 3D, Wetzlar, Germany).

The nanoscratch tests were performed on Tribology Test Equipment (CETR UNMT-1, CETR, Campbell, United States). The diamond tip with the radius of 2.5 μm was set to generate a series of 2 mm-long wear tracks on film surface at a constant velocity of 12.5 μm/s, with the load increasing linearly from 2 mN to 50 mN. The scratch morphology was observed using a microscope (Leica DCM 3D, Wetzlar, Germany).

The wear resistance tests were performed on Tribology Test Equipment (CETR UNMT-1, CETR, Campbell, CA, USA). At room temperature, a reciprocating wear test was conducted using an Al_2_O_3_ grinding ball with a diameter of 1.5 mm. The applied load was 20 mN, the reciprocating friction distance was 1 cm, and the duration time was 15 min. The surface friction coefficient was recorded, while the wear tracks were observed using scanning electron microscopy (S-3400N, Hitachi, Tokyo, Japan).

## 3. Results and Discussion

### 3.1. Effect of Ni on the Phase Structure

TiB_2_ films with different nickel contents were deposited using radio frequency magnetron sputtering. The film composition obtained using EDS analysis are shown in Table 1. As the Ni target power increased, the Ni content ranged from 0 to 32.2 at.%. The increase in power applied to the nickel target leads to the back-sputtering of the lighter B element from the substrate film, resulting in a relative decrease in the B element within the coating.

**Figure 2:**
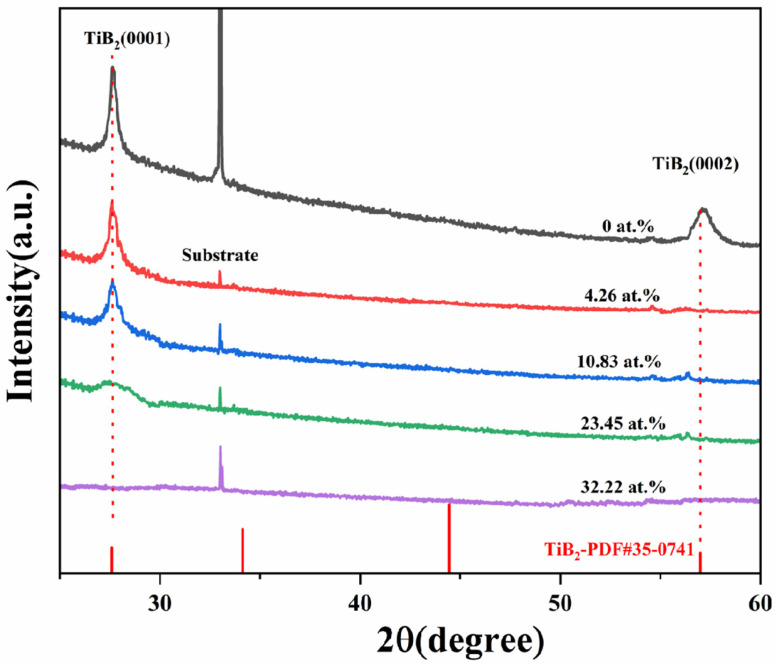
X-ray diffraction patterns of TiB_2_ films with different Ni contents.

In Figure 2, the X-ray diffraction patterns of TiB_2_ films with different Ni contents are presented. The findings indicate that all films were polycrystalline, with peaks corresponding exclusively to the hexagonal TiB_2_ phase (PDF#35-0741), and no crystalline Ni or other Ni-containing phases were identified. In comparison to the standard card of hexagonal TiB_2_, diffraction peaks of (0001) and (0002) were observed. It was noted that when nickel was incorporated into the films, the diffraction peak of (0002) disappeared. The growth of the film is influenced by surface energy, elastic strain energy, and interfacial energy. When the dominant factor is surface energy, the film preferentially grows along crystal faces with lower surface energy, and the (0001) crystal face exhibits the lowest surface energy for TiB_2_ film. The incorporation of nickel (4.26–23.45 at.%) did not influence the favored alignment of the TiB_2_ coating, but there was a gradual decrease in intensity and broadening of the (0001) diffraction peaks of TiB_2_ phase. When the nickel content increased to 32.22%, the diffraction pattern became nearly linear, indicating that the addition of Ni impeded the growth of TiB_2_ grains and resulted in a reduction in their size and an amorphous tendency. The results indicate that the presence of nickel hinders the nucleation and growth of TiB_2_ grains, and this hindrance becomes more pronounced as the nickel content increases. The faster diffusion rate of nickel atoms in the hexagonal lattice may account for this phenomenon. As the nickel content increases, an increasing number of nickel atoms are enriched in the grain boundary, which hinders the growth of grain. No crystalline Ni or other Ni-containing phase could be identified, indicating that nickel was present in an amorphous phase. Overall, these results provide valuable insights regarding how varying levels of nickel content can impact structural characteristics within TiB_2_ films.

**Figure 3:**
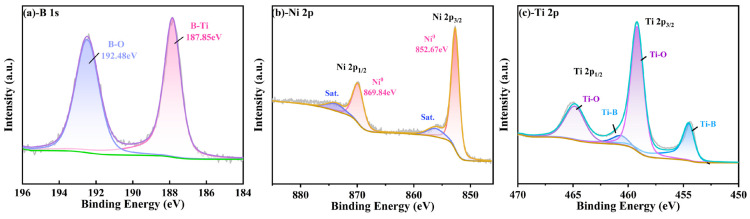
XPS spectra of B, Ni Ti, obtained from the surface of TiB_2_ film with 4.26 at.% Ni (**a**) -B 1s; (**b**) -Ni 2p; (**c**) -Ti 2p.

The deposited TiB_2_ coating was subjected to X-ray photoelectron spectroscopy analysis in order to determine the chemical elements present and their respective bonding states. Firstly, the surfaces of spectral lines of B 1s, Ni 2p and Ti 2p were analyzed, and the results of the TiB_2_-Ni (4.26 at.%) film are presented in Figure 3. The spectra were calibrated for the value of carbon peak C 1 s at 284.8 eV, which was used as a reference. In Figure 3a, the B-Ti bond was observed at 187.85 eV, corresponding to the presence of TiB_2_. Figure 3b depicts the Ni 2p spectrum of the TiB_2_-Ni film, and the Ni-Ni bond was identified at the binding energies of 869.84 eV and 852.67 eV. The Ti 2p region is considerably more complex due to the spin–orbit splitting and higher electron yield of titanium, which results in a higher background step and more prominent plasmon peaks [37]. As given in Figure 3c, it can be seen that the Ti-B bond can be fitted at 454.51 eV and 460.67 eV by dividing the peaks of Ti2p_2/3_ and Ti2p_1/2_, which correspond to TiB_2_. In the meantime, the Ti-Ni peak cannot be fitted from Ti2p spectra, and no peak for B-Ni bonding could be fitted from the B 1s spectrum.

**Figure 4:**
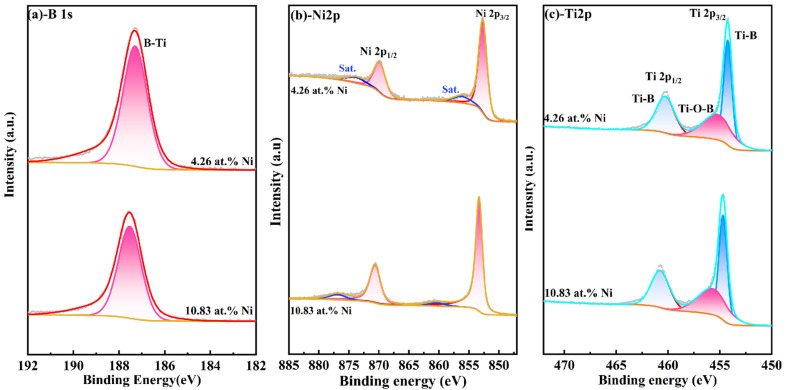
XPS spectra of TiB_2_-Ni films with different nickel content after etching 120 s (**a**) -B 1s; (**b**) -Ni 2p; (**c**) -Ti 2p.

Additionally, the B-O bond peak was detected at the position of 192.48 eV in the B 1S map (Figure 3a), and the Ti-O bond peak was also detected in the Ti-2p map, which indicates that element boron and titanium samples are usually oxidized at the surface. To avoid the impact of surface contaminants on the results, the sample surface was etched using Ar+ for 120 s, and Figure 4 shows the XPS spectra of TiB_2_-Ni films with different nickel content. As shown in Figure 4a, the B-O bond peak was not detected again; moreover, the oxygen-containing bond in the coatings was significantly weakened. It also can be seen that the valence state of elements in the coatings does not change significantly with the addition of nickel content.

The findings indicate that there is no chemical bonding between Ni and Ti/B atoms within the film, which demonstrates that the main chemical form of the Ni is the metallic state. And the presence of Ti-O and B-O in XPS results may be related to the residual oxygen in the vacuum chamber, since no oxide phases of Ti and B were found in XRD spectra.

**Figure 5:**
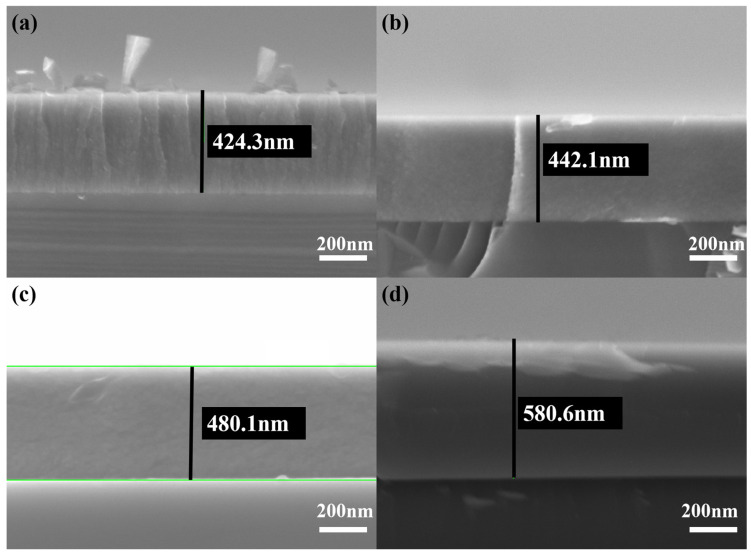
Cross-sectional SEM micrographs of TiB_2_ film with different Ni contents: (**a**) 4.26 at.%; (**b**) 10.83 at.%; (**c**) 23.45 at.%; (**d**) 32.22 at.%.

Figure 5 depicts a cross-sectional SEM micrograph of a TiB_2_ film with varying Ni contents. It is evident from Figure 5 that there is an absence of any voids or gaps between the film and substrate, which indicates a good adhesion between the coating and substrate. The columnar crystals exhibit a typical growth structure when the nickel content is 4.26%, as shown in Figure 5a. With the increase in nickel content, the columnar crystals within the coating are gradually eliminated, the fine grains undergo deformation, and the overall columnar structure is progressively refined.

**Figure 6:**
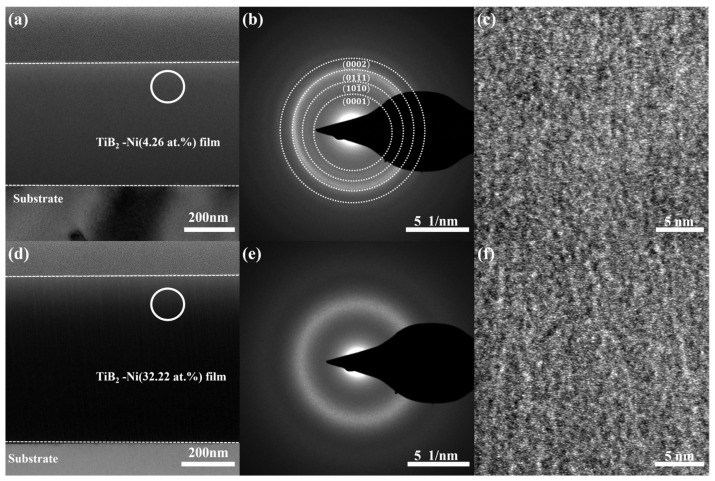
TEM results for TiB_2_ films with different Ni contents: (**a**) bright-field cross-sectional image (4.26 at.%); (**b**) SAED pattern from the area marked as circle in (**a**); (**c**) HRTEM image; (**d**) bright-field cross-sectional image (32.22 at.%); (**e**) SAED pattern from the area marked as circle in (**d**); (**f**) HRTEM image.

The transmission electron microscopy (TEM) results for TiB_2_ films with nickel content of 4.26 at.% and 32.22 at.% are presented in Figure 6. The bright-field cross-sectional images of the films are shown in Figure 6a,d, revealing a predominant presence of small columnar grains in the film microstructure. At a nickel content of 4.26 at.%, the selected area diffraction pattern (Figure 6b) exhibits a polycrystalline diffraction ring, and the reflection peaks such as (0001) are observed without any evidence of crystalline Ni phase, suggesting the existence of a small amount of TiB_2_ nanocrystals and amorphous nickel within the film structure. With an increase in nickel content to 32.22 at.%, there is an observable broadening of the electron diffraction ring (Figure 6e), indicating that the composite film is predominantly amorphous. The combination of the local high-resolution image (Figure 6c,f) reveals a homogeneous structure in the film, wherein the addition of nickel hinders the growth of TiB_2_ grains, resulting in a tendency towards amorphousness. The microstructure of TiB_2_-Ni films was significantly influenced by the Ni content. Increasing the Ni content led to grain refinement and a more compact and denser film structure. It should be noted that no crystalline Ni phase was observed in any of the films, regardless of their Ni contents.

**Figure 7:**
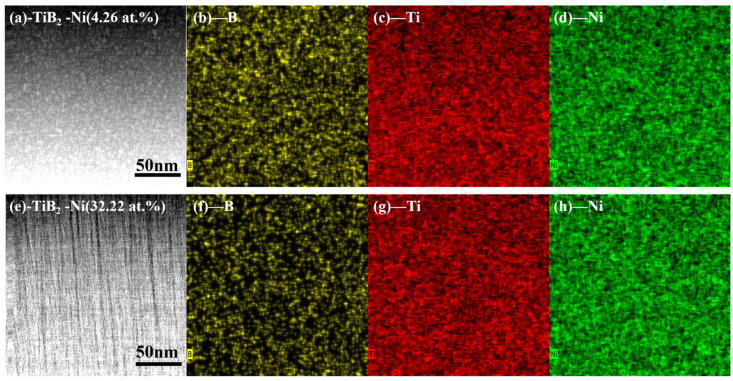
HAADF images and the corresponding EDX map scan of B, Ti and Ni elements of TiB_2_-Ni films. (**a**) Image of TiB_2_ with 4.26 at.% Ni film; (**b**) map scan of B; (**c**) map scan of Ti; (**d**) map scan of Ni; (**e**) Image of TiB_2_ with 32.22 at.% Ni film; (**f**) map scan of B; (**g**) map scan of Ti; (**h**) map scan of Ni.

Figure 7 presents the high-angle annual dark-field (HAADF) image of TiB_2_-Ni (4.26 and 32.22 at.%), along with the element mapping scans for B, Ti and Ni. As depicted in Figure 7b–d, the distribution of nickel in the TiB_2_ coating is uniform with a nickel content of 4.26 at.% (Figure 7a). When the nickel content increased to 32.22 at.% (Figure 7d), the distribution of B element became limited. With the increase in the nickel target power, the more B atoms on the surface of the substrate are sputtered, resulting in a decrease in element B, and the amorphous-phase Ni atoms can be randomly dispersed within the coatings.

**Figure 8:**
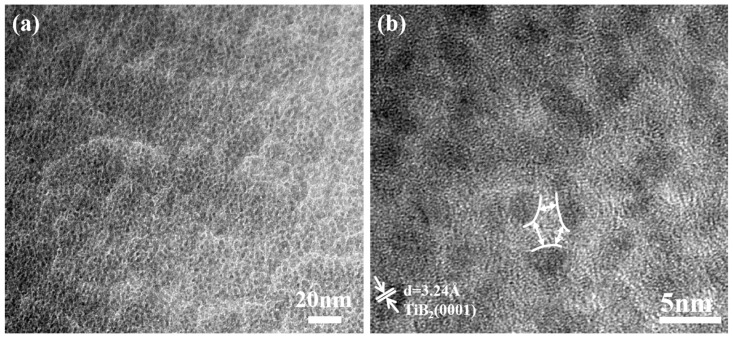
Plan-view TEM micrograph of TiB_2_ with 10.83 at.% Ni coating. (**a**) low-magnification; (**b**) high-magnification.

The TEM observations combined with the XPS results and XRD results verified that the Ni atoms existed in films mainly as an amorphous phase. To further investigate the relationship between nickel and titanium diboride in the coatings, the surface of the composite coating was characterized using transmission electron microscopy. Figure 8 shows the plan-view TEM micrographs of TiB_2_ with 10.83 at.% Ni coating. It can be seen from the figure that the coating has a two-phase structure with white bright bands surrounding dark particles (Figure 8a). Further analysis (Figure 8b) reveals that the dark particles are TiB_2_ nanocrystals with a grain size ranging from 3 to 5 nm, and nickel is present as an amorphous phase at the boundaries of the TiB_2_. The addition of nickel exists as a single phase, contributing to the development of a two-phase composite structure. The diffusion rate of nickel in the hexagonal lattice is significantly higher [33], facilitating its migration to the grain boundary of TiB_2_, thereby effectively impeding the grain growth of TiB_2_.

### 3.2. Effect of Ni on Mechanical Properties

**Figure 9:**
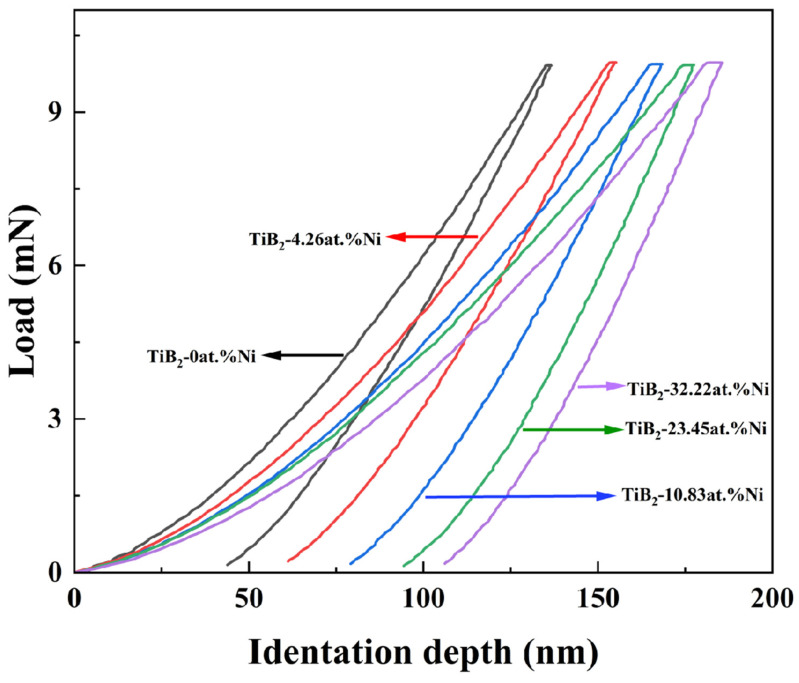
The indentation curves of TiB_2_ film with different Ni contents.

**Figure 10:**
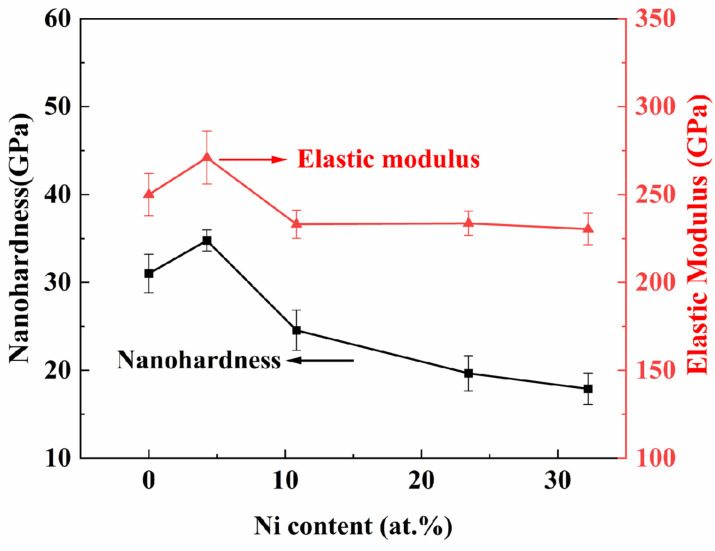
The nanohardness and elastic modulus of TiB_2_ film with different Ni contents.

Figure 9 illustrates the load–indentation-depth curves of TiB_2_ films with varying Ni contents. The loading and unloading curves of the films exhibit nonlinearity. Under the same load, the maximum indentation depth and the residual indentation depth gradually increase with the increase in nickel content. As shown in Figure 10, the results indicate that there is an initial increase followed by a subsequent decrease in nanohardness. The nanohardness of the TiB_2_ film is determined to be 31 ± 2.2 GPa. With the addition of nickel of 4.26 at.%, the nanohardness of the composite film reaches 34.8 ± 1.2 GPa. However, as the nickel content further increases to 32.22 at.%, a continuous decrease in nanohardness to 17.89 ± 1.8 GPa is observed for the composite film. As depicted in Figure 10, the curve of elastic modulus exhibits a similar trend to that of nanohardness. Specifically, the elastic modulus measures 271 ± 15 GPa, at a Ni content of 4.26 at.%. As the nickel content further increases, the elastic modulus of the film gradually decreases, reaching 230 ± 9 GPa at a nickel content of 32.22 at.%.

When the nickel content is below 10.83 at.%, the coating hardness remains at or above 20 GPa. On one hand, the optimal growth of TiB_2_ (0001) crystal surface helps improve the film hardness [34]. On the other hand, it may be attributed to the grain refinement effect of TiB_2_ and the inhibition of grain boundary sliding by the nickel amorphous phase [38]. The theoretical analysis in reference [32] also indicates that when the nickel content is below 13 at.%, the hardness of the coating exhibits a slight decrease; however, it remains comparable to that of the pure titanium diboride coating.

As the nickel content progressively increases, the hardness of the coatings decreases. An increase in nickel content results in a notable reduction in boron (B) concentration within the film, consequently diminishing the number of Ti-B and B-B bonds. The enrichment of nickel in the boundaries of titanium diboride increased with the increase in nickel element. Additionally, it is evident from the XRD pattern that the addition of nickel leads to a reduction in the crystallinity of titanium diboride, consequently altering the hardness of the coatings. Consequently, an increase in Ni content results in an increased load for the softer Ni phase to bear, consequently leading to a decrease in hardness for the composite film as well.

**Figure 11:**
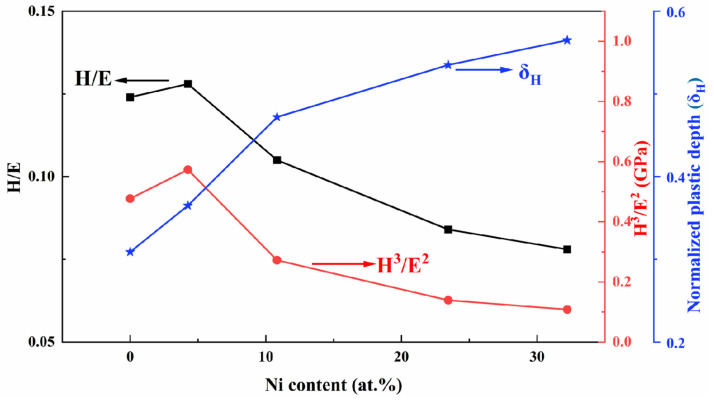
The values of H/E, H^3^/E^2^, normalized plastic depth value (δ_H_) of TiB_2_ film with different Ni contents.

With regard to the elastic behavior, it is not sufficient to describe the film properties solely based on hardness; instead, the ratio of hardness (H) to elastic modulus (E) should be considered [39]. The H/E ratio indicates the resistance to cracking, while the H^3^/E^2^ ratio reflects the resistance to plastic deformation [40]. Figure 11 illustrates the values of H/E and H^3^/E^2^ for TiB_2_ films with varying Ni contents. It can be observed that when the nickel content is 4.26 at.%, the values of H/E and H^3^/E^2^ reach their maximum at 0.128 and 0.573 GPa, respectively. When nickel is dissolved into the lattice of titanium diboride, it causes lattice distortion, resulting in an increase in the internal stress of the coating and an increase in the elastic modulus [41]. Previous studies [16,42] have indicated that a high value of H^3^/E^2^ along with a high ratio of H/E (>0.1), combined with a dense microstructure, are favorable for enhancing film toughness. However, when the nickel content exceeds 10.83%, the value of H/E drops below 0.1. Upon increasing nickel content from 10.83 at.% to 32.22 at.%, both ratios gradually decrease. The two values of H/E and H^3^/E^2^ reflect key parameters relating to film resistance against elastoplastic deformation, and demonstrate positive correlations with fracture toughness as well as wear resistance [43].

The normalized plastic depth value (δ_H_) is also utilized to assess the film’s plasticity, as illustrated in Figure 11. The plastic deformation capacity [44] serves as an indicator of toughness to a certain extent, with higher toughness associated with stronger plastic deformation capacity. It can be observed from Figure 10 that with an increase in Ni content from 0 at.% to 10.83 at.%, there is a large rise in the normalized plastic depth. As the content continues to increase, the rate of growth in the normalized plastic depth decelerates. With an increase in the depth of deformation, there is a corresponding enhancement in plasticity index, indicating greater plastic deformation ability and improved toughness.

**Figure 12:**
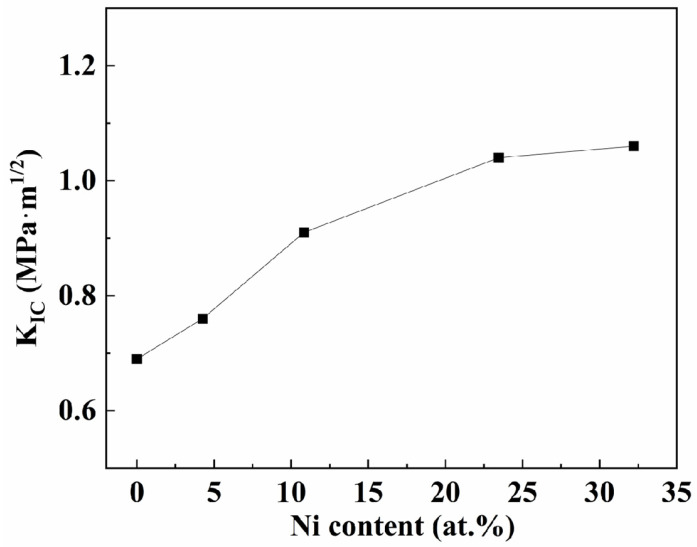
The calculated fracture toughness with different Ni contents.

The nanoindentation test has proven to be a powerful technique to evaluate material’s brittleness or resistance to fracture [45]. The calculated results of fracture toughness of the films with different nickel contents used by Equation (1) are given in Figure 12. As illustrated in the figure, when the nickel content increases to 10.83 at.%, the toughness of the coatings increases faster. The TiB_2_ film exhibits a fracture toughness of 0.69 MPa·m^1/2^, and this value increases to 0.91 MPa·m^1/2^ when the nickel content reaches 10.83 at.%. When the nickel content increases further, the film toughness slows down, and the value increases to 1.06 MPa·m^1/2^ when the nickel content reaches 32.22 at.% in composite films. This is consistent with the trend of normalized plastic depth.

**Figure 13:**
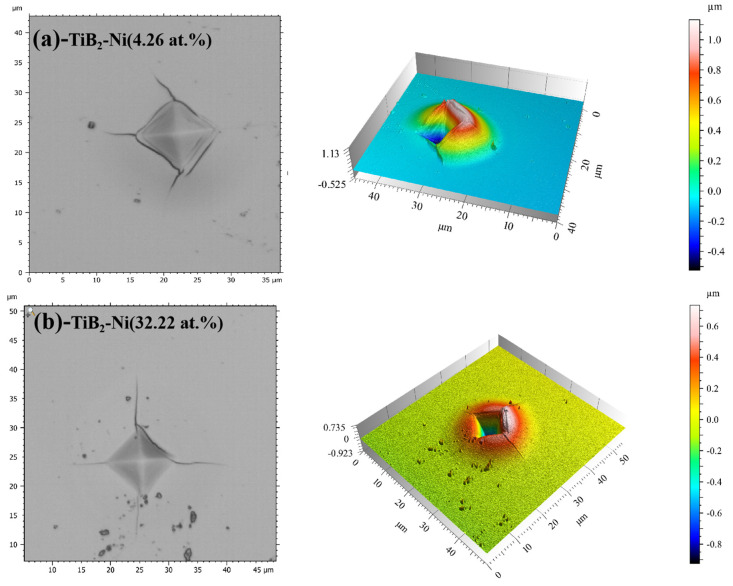
Indentation morphology of TiB_2_ film with different Ni contents (**a**) 4.26 at.%;(**b**) 32.22 at.%.

Figure 13 displays the indentation morphology of the TiB_2_ film with varying Ni contents (4.26 at.% and 32.22 at.%). It is evident that circular cracks appear around the indentation, indicating a tendency toward brittle fractures. The formation and propagation of these circular cracks are primarily attributed to macroscopic plastic deformation, which leads to crack propagation during continuous loading due to uncoordinated deformation between the film and substrate. However, as the Ni content increases, the presence and visibility of circular cracks decrease gradually under identical load conditions, suggesting an increase in the fracture toughness of films as well. Toughness refers to the film’s ability to absorb energy between deformation and fracture [46]. The increase in nickel content leads to a reduction in circumferential cracks of the coating under the same load, gradually diminishing until they disappear completely. Simultaneously, based on the three-dimensional indentation morphology (Figure 12), it is evident that an increase in nickel content leads to a more homogeneous deformation of the film. When the nickel content reached 4.26 at.%, annular cracks manifested both around and within the indentation, accompanied by non-uniform deformation. Under the same load, annular cracks did not appear inside the indentation, and the film deformation was uniform when the nickel content increased to 32.22 at.%. This observation suggests an enhancement in the toughness of the coating with the increase in nickel content. When crack propagation occurs in the films, the Ni metal phase exhibits excellent plastic deformation ability, effectively absorbing the energy of crack propagation. This hinders continuous crack propagation and ultimately improves the toughness of the TiB_2_-Ni composite film.

**Figure 14:**
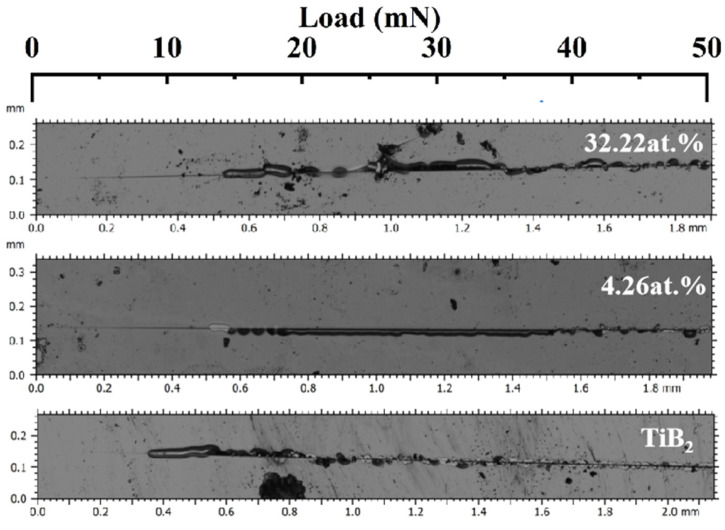
The nanoscratch morphology of TiB_2_-Ni coatings.

Scratch testing was also used to evaluate the toughness of hard coatings by the references [25,47]. Figure 14 shows the scratch morphology with different Ni contents. As shown in the figure, the titanium diboride coating exhibits brittle characteristics. Shortly after testing, it develops cracks and subsequently spalls, which is indicative of the inherent brittleness associated with ceramic coatings. The addition of nickel delays the cracking time of the film. Owing to the accumulation of nickel in the amorphous phase at the boundary of titanium diboride, the soft metal can effectively absorb the energy associated with plastic deformation, and the microcracks initiated in TiB_2_ are hindered by the surrounding nickel phase. The composite structure facilitates the passivation and deflection of cracks [48], thereby enhancing the toughness and binding force.

**Figure 15:**
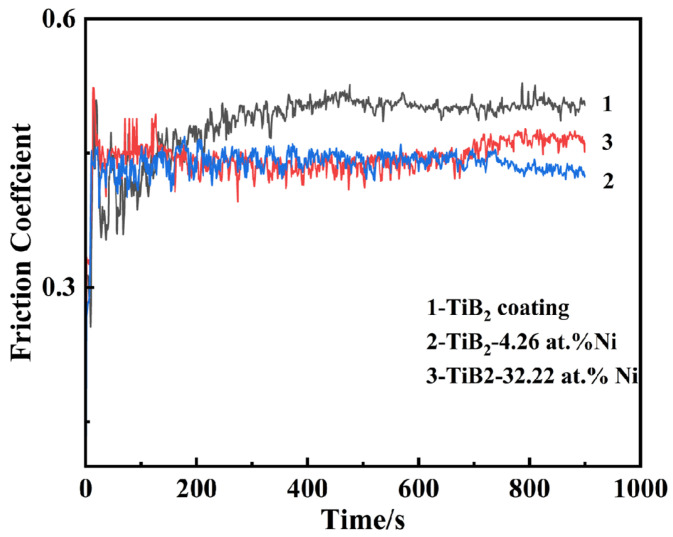
The friction coefficient of TiB_2_-Ni composite coatings.

**Figure 16:**
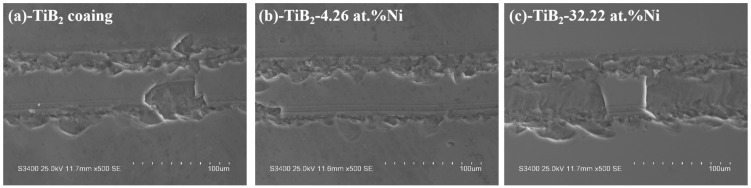
Wear track morphology of TiB_2_-Ni coatings. (**a**) TiB_2_ coating, (**b**)TiB_2_-4.26 at.% Ni coating, (**c**) TiB_2_-32.22 at.% Ni coating.

Figure 15 illustrates the friction coefficient curve of the TiB_2_-Ni composite coatings. For TiB_2_ films, the friction coefficient remained consistently around 0.5. Upon the addition of nickel, the friction coefficient exhibited significant fluctuations during the initial stage but subsequently stabilized. Specifically, when the nickel content was 4.26 at.%, the friction coefficient stabilized at approximately 0.4. As the nickel content increased further, the friction coefficient demonstrated a gradual upward trend during the later stages of friction. The wear track morphology is shown in Figure 16. Titanium diboride coatings exhibit brittle spalling during wear, primarily due to the inherent brittleness of TiB_2_ and insufficient adhesion between the coating and substrate. In contrast, a composite coating containing 4.26 at.% Ni demonstrated superior performance, exhibiting higher hardness, shallower wear marks, and reduced spalling compared to TiB_2_ coatings. This improvement was attributed to the formation of a two-phase structure of TiB_2_ particles surrounded by amorphous nickel. During plastic deformation under external forces, cracks were less likely to initiate at grain boundaries. The ductile nickel allowed it to absorb some of the deformation energy, effectively inhibiting crack initiation. Even if cracks did form, their propagation was hindered by mechanisms such as bypassing or cutting through the coating, further preventing crack extension. However, when the nickel content increased to 32.22 at.%, the coating’s wear resistance significantly decreased, with evident plastic deformation observed in the wear tracks. This degradation in performance was likely due to the substantial reduction in hardness caused by the higher nickel content.

## 4. Conclusions

In this paper, TiB_2_-Ni films were deposited using radio frequency magnetron sputtering. The study focused on the impact of Ni content on the composition, microstructure, and mechanical properties of the films. It was observed that as the radio frequency power of target Ni increased from 5 to 30 W, the nickel content in TiB_2_ films also increased from 4.26 at.% to 32.22 at.%. The increase in Ni content led to grain refinement and a more compact and denser film structure. Diffraction peaks of (0001) for hexagonal TiB_2_ were detected. With the addition of nickel content, the transition of titanium diboride to an amorphous state commenced, and no crystalline Ni or other Ni-containing phase was detected. Nickel existed at the boundary of titanium diboride as an amorphous phase, which formed a structure of titanium diboride encapsulated by the nickel layer. When the amount of nickel added is below 10.83 at%, the hardness of the coating can be maintained above 20 GPa. At a nickel content of 4.26 at.%, nanohardness was measured at approximately 34.8 ± 1.2 GPa, while elastic modulus was approximately 271 ± 15 GPa with high ratios of H/E and H^3^/E^2^. The enrichment of nickel in the grain boundary hindered the growth of titanium diboride and was beneficial to the enhancement of coating hardness. When the nickel content increased further, it became increasingly concentrated at the boundary, and titanium diboride tended to amorphous, which resulted in a decrease in the hardness of the film. The fracture toughness increased from 0.69 MPa·m^1/2^ to 1.06 MPa·m^1/2^ when the nickel content changed from 0 to 32.22 at.% in composite films. The addition of nickel enhanced the adhesion between the film and substrate while reducing the friction coefficient of the film. The Ni phase segregated in the boundary was found to prevent crack propagation through its superior plastic deformation ability, to achieve a toughening effect.

## Figures and Tables

**Table 1:** The element composition of TiB_2_ films with different Ni contents.

The Power on Ni Target/W	At.%			Ni/Ti
	Ni	Ti	B	
0	-	22.20	77.80	-
5	4.26	22.23	73.51	0.19
10	10.83	25.23	63.94	0.43
20	23.45	22.75	53.80	1.03
30	32.22	19.53	48.25	1.65

## Data Availability

The authors confirm that the data supporting the findings of this study are available within the article.
